# 527 Practical Use of Biomarkers During Nutritional Support of Pediatric Burn Patients

**DOI:** 10.1093/jbcr/irac012.157

**Published:** 2022-03-23

**Authors:** Jennifer Shiel, Kathy Prelack

**Affiliations:** Shriners Hospital for Children-Boston, Boston, Massachusetts; Shriners Hospitals for Children-Boston, Boston, Massachusetts

## Abstract

**Introduction:**

Current guidelines for evaluating nutritional risk in acutely ill patients incorporate the assessment of inflammation and disease burden. Inclusion of laboratory measures such as C-reactive protein (CRP) in screening criteria used to identify nutritional risk has gained credibility. Other biomarkers influenced by inflammation include visceral proteins albumin (ALB) and pre-albumin (PAB). While visceral proteins are not valid indicators of nutritional status, they may be indicators of nutritional risk, which could potentially lead to poor clinical outcome. The purpose of this study is to evaluate if PAB, ALB, CRP, are useful markers for predicting clinical outcomes in children with severe burn injury.

**Methods:**

As part of our quality assurance program, we collect data on all nutrition support interventions, monitoring and outcome in children admitted to our hospital with significant burn injuries. This analysis describes data collected from 2006-2019 in children who had a burn injury and received nutritional support for five days or greater. Data elements collected include general demographics, weekly measures PAB, CRP, ALB; length of stay (LOS), number of intensive care unit (ICU) days, days to wound closure, and days on nutrition support. Biomarkers PAB, ALB, CRP and burn size (as an indicator of disease burden) were entered into a multiple regression model using a stepwise procedure for each dependent outcome variables (LOS, ICU LOS, Days to Wound Closure, and Days on Nutrition Support).

**Results:**

A total of 182 patients, 7.0 ± 5.0 years of age with 41.1 ±16.9 % total body surface area (TBSA) burns were included in the analysis. TBSA, mean CRP and mean PAB were significant predictors of hospital LOS (R=0.60; p < 0.001); TBSA and PAB were significant predicters of ICU LOS (R=0.67; p< 0.000), days to wound healing (R= 0.37; p < 0.000) and days on nutrition support (R=0.60; p< 0.000). Albumin was not a significant predictor for any of the clinical outcome measures.

**Conclusions:**

Our findings indicate that monitoring PAB and CRP is useful for identifying risk of poor outcome. Mean PAB was inversely associated with LOS, ICU LOS, days to wound healing, and days on nutritional support even when controlling for CRP and burn size.

